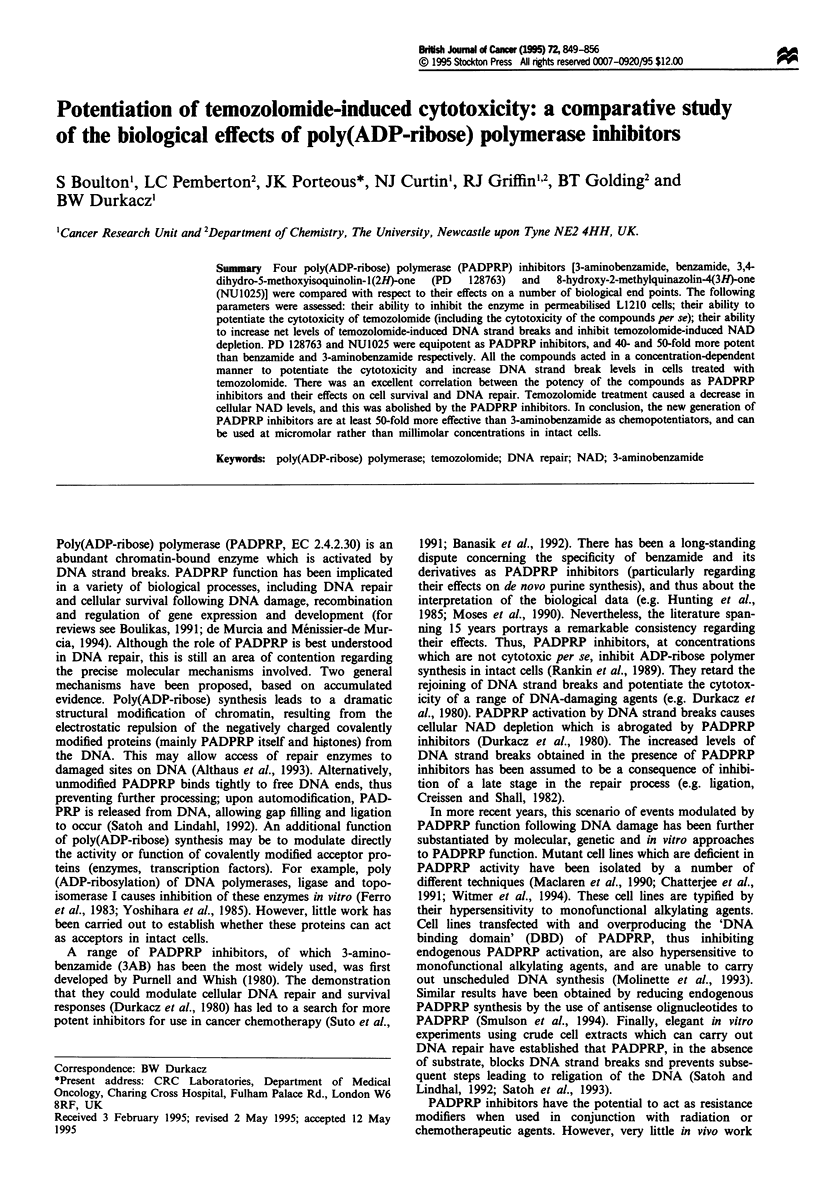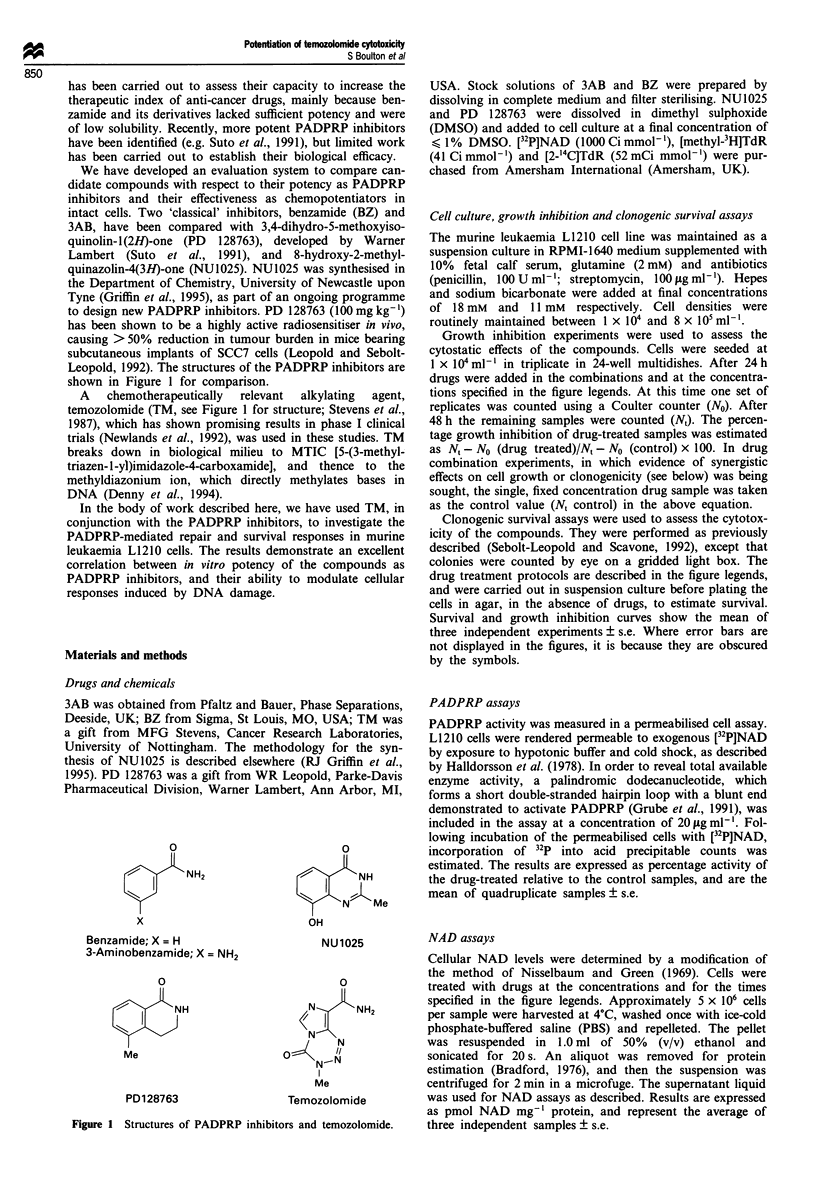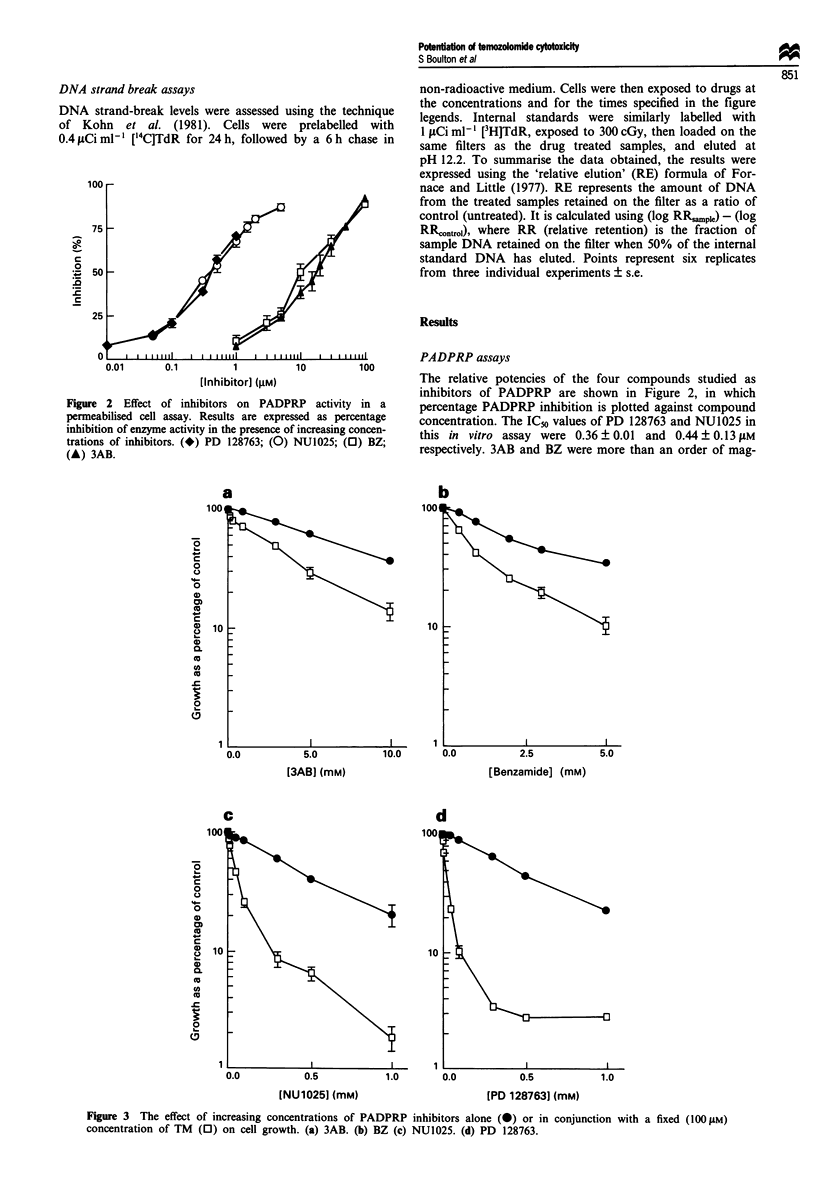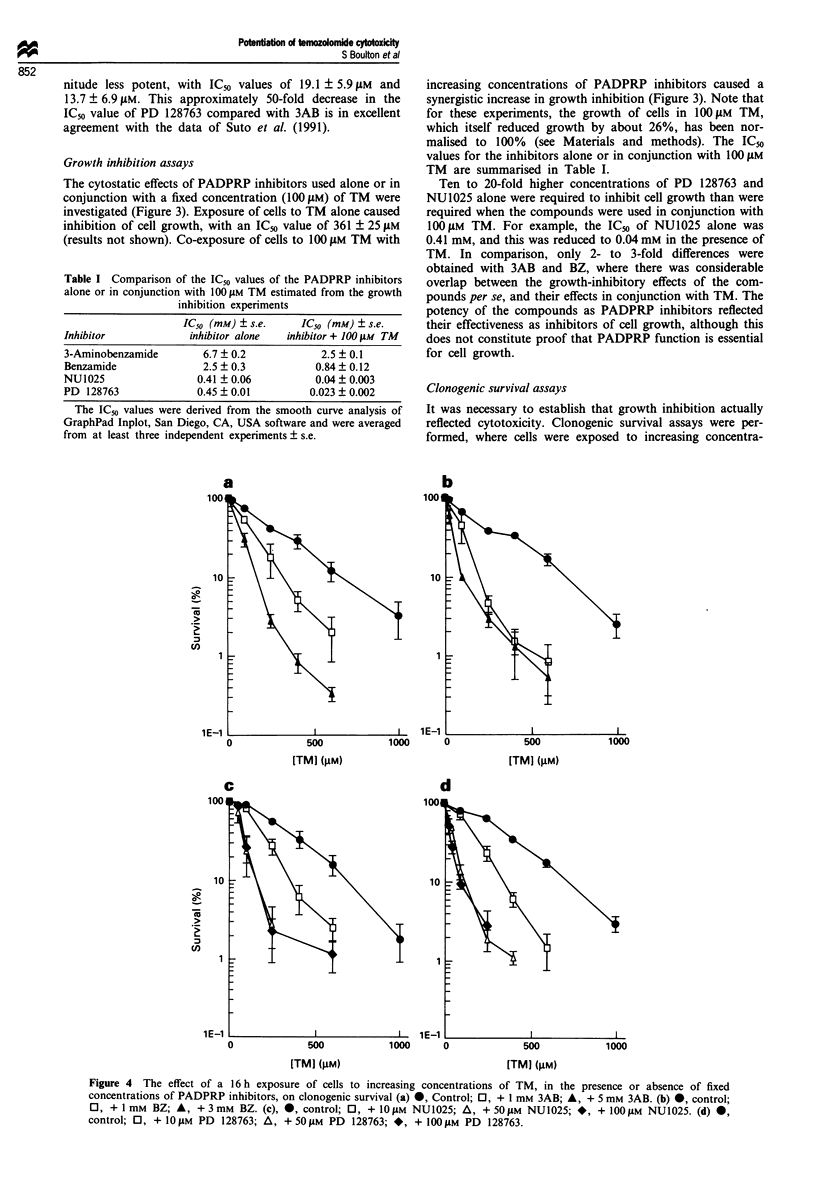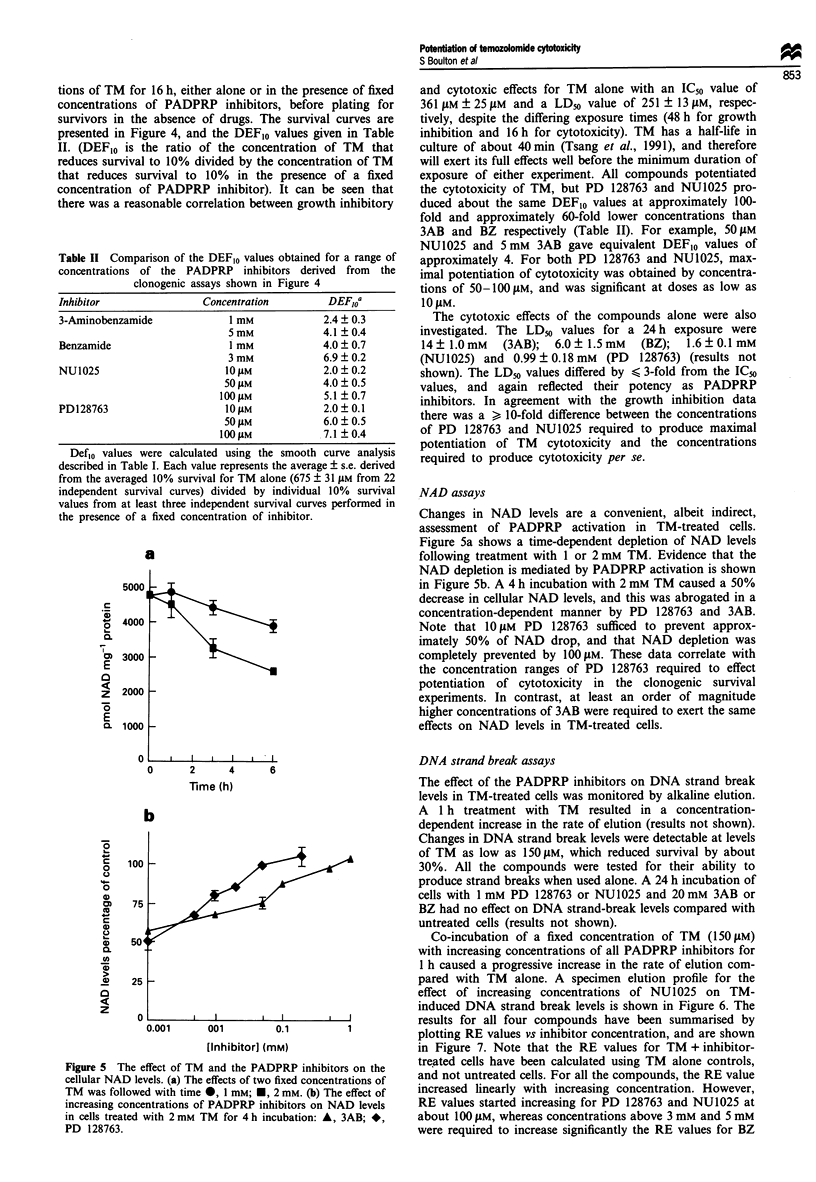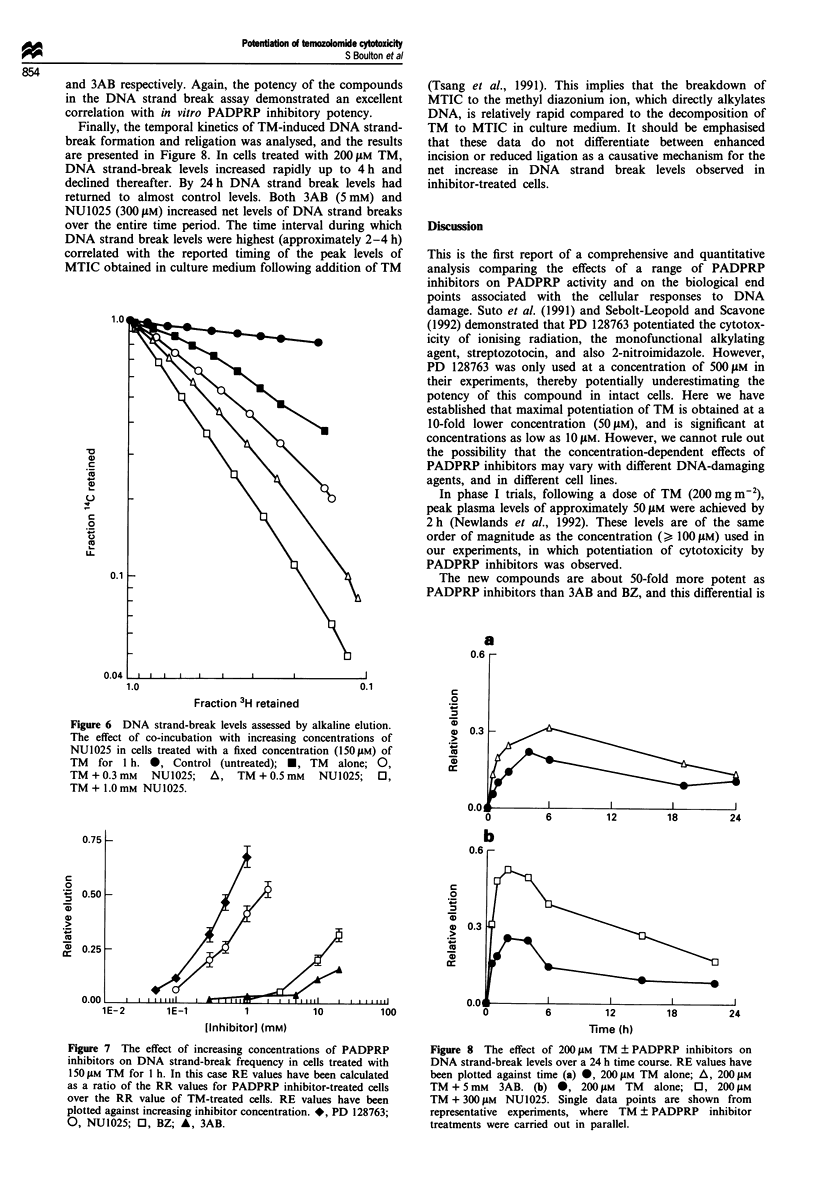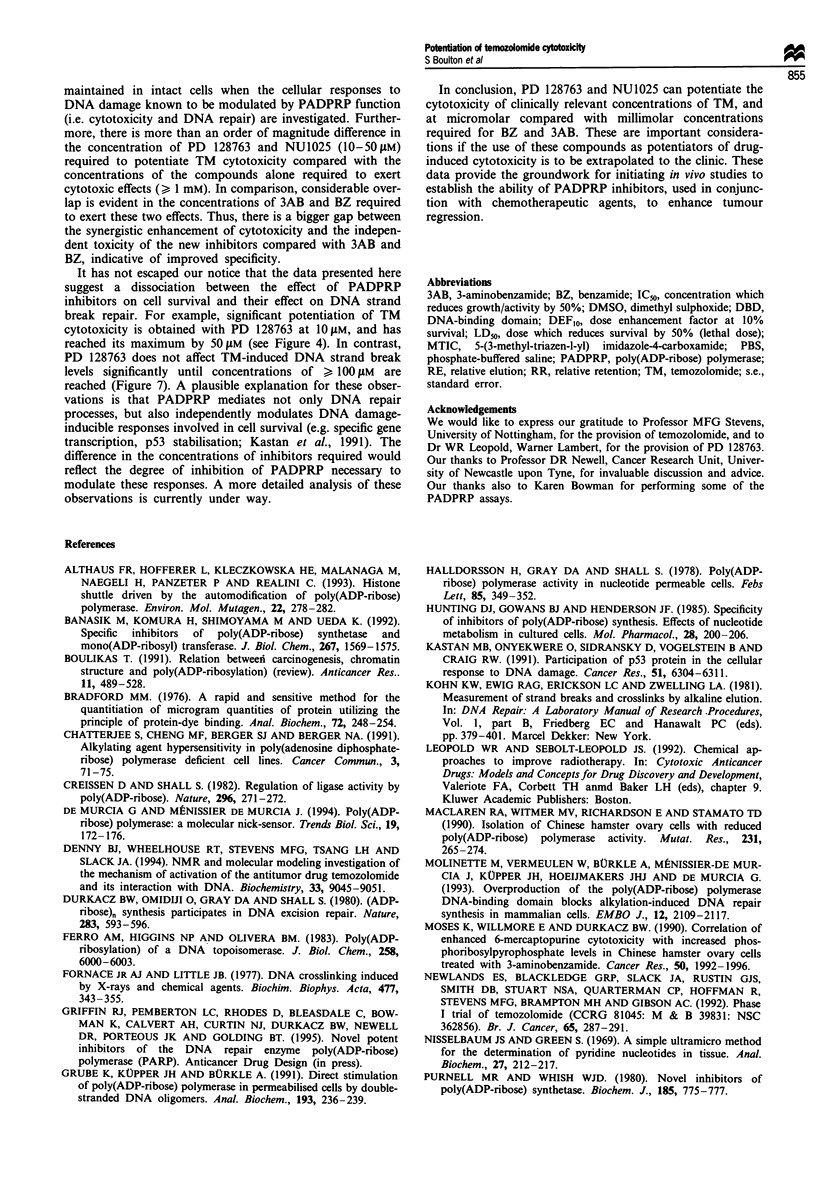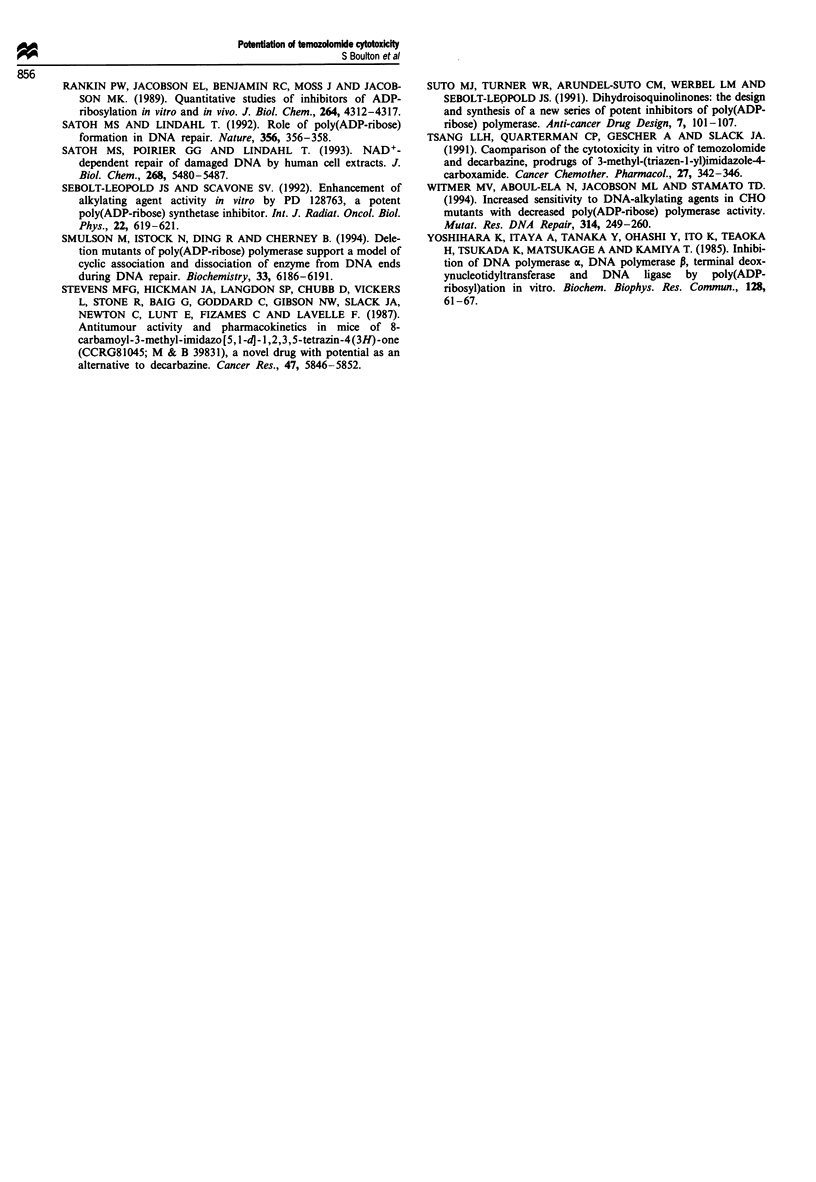# Potentiation of temozolomide-induced cytotoxicity: a comparative study of the biological effects of poly(ADP-ribose) polymerase inhibitors.

**DOI:** 10.1038/bjc.1995.423

**Published:** 1995-10

**Authors:** S. Boulton, L. C. Pemberton, J. K. Porteous, N. J. Curtin, R. J. Griffin, B. T. Golding, B. W. Durkacz

**Affiliations:** Cancer Research Unit, University, Newcastle upon Tyne, UK.

## Abstract

Four poly(ADP-ribose) polymerase (PADPRP) inhibitors [3-aminobenzamide, benzamide, 3,4-dihydro-5-methoxyisoquinolin-1(2H)-one (PD 128763) and 8-hydroxy-2-methylquinazolin-4(3H)-one (NU1025)] were compared with respect to their effects on a number of biological end points. The following parameters were assessed: their ability to inhibit the enzyme in permeabilised L1210 cells; their ability to potentiate the cytotoxicity of temozolomide (including the cytotoxicity of the compounds per se); their ability to increase net levels of temozolomide-induced DNA strand breaks and inhibit temozolomide-induced NAD depletion. PD 128763 and NU1025 were equipotent as PADPRP inhibitors, and 40- and 50-fold more potent than benzamide and 3-aminobenzamide respectively. All the compounds acted in a concentration-dependent manner to potentiate the cytotoxicity and increase DNA strand break levels in cells treated with temozolomide. There was an excellent correlation between the potency of the compounds as PADPRP inhibitors and their effects on cell survival and DNA repair. Temozolomide treatment caused a decrease in cellular NAD levels, and this was abolished by the PADPRP inhibitors. In conclusion, the new generation of PADPRP inhibitors are at least 50-fold more effective than 3-aminobenzamide as chemopotentiators, and can be used at micromolar rather than millimolar concentrations in intact cells.


British Journal of Cancer (1995) 72, 849–856

© 1995 Stockton Press All rights reserved 0007–0920/95 $12.00

### Potentiation of temozolomide-induced cytotoxicity: a comparative study of the biological effects of poly(ADP-ribose) polymerase inhibitors

S Boulton[1], LC Pemberton[2], JK Porteous*, NJ Curtin[1], RJ Griffin[1,2], BT Golding[2] and BW Durkacz[1]

[1]*Cancer Research Unit and* [2]*Department of Chemistry, The University, Newcastle upon Tyne NE2 4HH, UK.*

**Summary** Four poly(ADP-ribose) polymerase (PADPRP) inhibitors [3-aminobenzamide, benzamide, 3,4-dihydro-5-methoxyisoquinolin-1(2*H*)-one (PD 128763) and 8-hydroxy-2-methylquinazolin-4(3*H*)-one (NU1025)] were compared with respect to their effects on a number of biological end points. The following parameters were assessed: their ability to inhibit the enzyme in permeabilised L1210 cells; their ability to potentiate the cytotoxicity of temozolomide (including the cytotoxicity of the compounds *per se*); their ability to increase net levels of temozolomide-induced DNA strand breaks and inhibit temozolomide-induced NAD depletion. PD 128763 and NU1025 were equipotent as PADPRP inhibitors, and 40- and 50-fold more potent than benzamide and 3-aminobenzamide respectively. All the compounds acted in a concentration-dependent manner to potentiate the cytotoxicity and increase DNA strand break levels in cells treated with temozolomide. There was an excellent correlation between the potency of the compounds as PADPRP inhibitors and their effects on cell survival and DNA repair. Temozolomide treatment caused a decrease in cellular NAD levels, and this was abolished by the PADPRP inhibitors. In conclusion, the new generation of PADPRP inhibitors are at least 50-fold more effective than 3-aminobenzamide as chemopotentiators, and can be used at micromolar rather than millimolar concentrations in intact cells.

**Keywords:** poly(ADP-ribose) polymerase; temozolomide; DNA repair; NAD; 3-aminobenzamide

Poly(ADP-ribose) polymerase (PADPRP, EC 2.4.2.30) is an abundant chromatin-bound enzyme which is activated by DNA strand breaks. PADPRP function has been implicated in a variety of biological processes, including DNA repair and cellular survival following DNA damage, recombination and regulation of gene expression and development (for reviews see Boulikas, 1991; de Murcia and Ménissier-de Murcia, 1994). Although the role of PADPRP is best understood in DNA repair, this is still an area of contention regarding the precise molecular mechanisms involved. Two general mechanisms have been proposed, based on accumulated evidence. Poly(ADP-ribose) synthesis leads to a dramatic structural modification of chromatin, resulting from the electrostatic repulsion of the negatively charged covalently modified proteins (mainly PADPRP itself and histones) from the DNA. This may allow access of repair enzymes to damaged sites on DNA (Althaus *et al.*, 1993). Alternatively, unmodified PADPRP binds tightly to free DNA ends, thus preventing further processing; upon automodification, PADPRP is released from DNA, allowing gap filling and ligation to occur (Satoh and Lindahl, 1992). An additional function of poly(ADP-ribose) synthesis may be to modulate directly the activity or function of covalently modified acceptor proteins (enzymes, transcription factors). For example, poly (ADP-ribosylation) of DNA polymerases, ligase and topoisomerase I causes inhibition of these enzymes *in vitro* (Ferro *et al.*, 1983; Yoshihara *et al.*, 1985). However, little work has been carried out to establish whether these proteins can act as acceptors in intact cells.

A range of PADPRP inhibitors, of which 3-aminobenzamide (3AB) has been the most widely used, was first developed by Purnell and Whish (1980). The demonstration that they could modulate cellular DNA repair and survival responses (Durkacz *et al.*, 1980) has led to a search for more potent inhibitors for use in cancer chemotherapy (Suto *et al.*, 1991; Banasik *et al.*, 1992). There has been a long-standing dispute concerning the specificity of benzamide and its derivatives as PADPRP inhibitors (particularly regarding their effects on *de novo* purine synthesis), and thus about the interpretation of the biological data (e.g. Hunting *et al.*, 1985; Moses *et al.*, 1990). Nevertheless, the literature spanning 15 years portrays a remarkable consistency regarding their effects. Thus, PADPRP inhibitors, at concentrations which are not cytotoxic *per se*, inhibit ADP-ribose polymer synthesis in intact cells (Rankin *et al.*, 1989). They retard the rejoining of DNA strand breaks and potentiate the cytotoxicity of a range of DNA-damaging agents (e.g. Durkacz *et al.*, 1980). PADPRP activation by DNA strand breaks causes cellular NAD depletion which is abrogated by PADPRP inhibitors (Durkacz *et al.*, 1980). The increased levels of DNA strand breaks obtained in the presence of PADPRP inhibitors has been assumed to be a consequence of inhibition of a late stage in the repair process (e.g. ligation, Creissen and Shall, 1982).

In more recent years, this scenario of events modulated by PADPRP function following DNA damage has been further substantiated by molecular, genetic and *in vitro* approaches to PADPRP function. Mutant cell lines which are deficient in PADPRP activity have been isolated by a number of different techniques (Maclaren *et al.*, 1990; Chatterjee *et al.*, 1991; Witmer *et al.*, 1994). These cell lines are typified by their hypersensitivity to monofunctional alkylating agents. Cell lines transfected with and overproducing the 'DNA binding domain' (DBD) of PADPRP, thus inhibiting endogenous PADPRP activation, are also hypersensitive to monofunctional alkylating agents, and are unable to carry out unscheduled DNA synthesis (Molinette *et al.*, 1993). Similar results have been obtained by reducing endogenous PADPRP synthesis by the use of antisense olignucleotides to PADPRP (Smulson *et al.*, 1994). Finally, elegant *in vitro* experiments using crude cell extracts which can carry out DNA repair have established that PADPRP, in the absence of substrate, blocks DNA strand breaks snd prevents subsequent steps leading to religation of the DNA (Satoh and Lindhal, 1992; Satoh *et al.*, 1993).

PADPRP inhibitors have the potential to act as resistance modifiers when used in conjunction with radiation or chemotherapeutic agents. However, very little *in vivo* work

Correspondence: BW Durkacz
*Present address: CRC Laboratories, Department of Medical Oncology, Charing Cross Hospital, Fulham Palace Rd., London W6 8RF, UK
Received 3 February 1995; revised 2 May 1995; accepted 12 May 1995

has been carried out to assess their capacity to increase the therapeutic index of anti-cancer drugs, mainly because benzamide and its derivatives lacked sufficient potency and were of low solubility. Recently, more potent PADPRP inhibitors have been identified (e.g. Suto *et al.*, 1991), but limited work has been carried out to establish their biological efficacy.

We have developed an evaluation system to compare candidate compounds with respect to their potency as PADPRP inhibitors and their effectiveness as chemopotentiators in intact cells. Two 'classical' inhibitors, benzamide (BZ) and 3AB, have been compared with 3,4-dihydro-5-methoxyisoquinolin-1(2*H*)-one (PD 128763), developed by Warner Lambert (Suto *et al.*, 1991), and 8-hydroxy-2-methylquinazolin-4(3*H*)-one (NU1025). NU1025 was synthesised in the Department of Chemistry, University of Newcastle upon Tyne (Griffin *et al.*, 1995), as part of an ongoing programme to design new PADPRP inhibitors. PD 128763 (100 mg $kg^{-1}$) has been shown to be a highly active radiosensitiser *in vivo*, causing >50% reduction in tumour burden in mice bearing subcutaneous implants of SCC7 cells (Leopold and Sebolt-Leopold, 1992). The structures of the PADPRP inhibitors are shown in Figure 1 for comparison.

A chemotherapeutically relevant alkylating agent, temozolomide (TM, see Figure 1 for structure; Stevens *et al.*, 1987), which has shown promising results in phase I clinical trials (Newlands *et al.*, 1992), was used in these studies. TM breaks down in biological milieu to MTIC [5-(3-methyltriazen-1-yl)imidazole-4-carboxamide], and thence to the methyldiazonium ion, which directly methylates bases in DNA (Denny *et al.*, 1994).

In the body of work described here, we have used TM, in conjunction with the PADPRP inhibitors, to investigate the PADPRP-mediated repair and survival responses in murine leukaemia L1210 cells. The results demonstrate an excellent correlation between *in vitro* potency of the compounds as PADPRP inhibitors, and their ability to modulate cellular responses induced by DNA damage.

### Materials and methods

### *Drugs and chemicals*

3AB was obtained from Pfaltz and Bauer, Phase Separations, Deeside, UK; BZ from Sigma, St Louis, MO, USA; TM was a gift from MFG Stevens, Cancer Research Laboratories, University of Nottingham. The methodology for the synthesis of NU1025 is described elsewhere (RJ Griffin *et al.*, 1995). PD 128763 was a gift from WR Leopold, Parke-Davis Pharmaceutical Division, Warner Lambert, Ann Arbor, MI, USA. Stock solutions of 3AB and BZ were prepared by dissolving in complete medium and filter sterilising. NU1025 and PD 128763 were dissolved in dimethyl sulphoxide (DMSO) and added to cell culture at a final concentration of ⩽1% DMSO. [$^{32}$P]NAD (1000 Ci $mmol^{-1}$), [methyl-$^{3}$H]TdR (41 Ci $mmol^{-1}$) and [2-$^{14}$C]TdR (52 mCi $mmol^{-1}$) were purchased from Amersham International (Amersham, UK).

**Figure 1** Structures of PADPRP inhibitors and temozolomide.

### *Cell culture, growth inhibition and clonogenic survival assays*

The murine leukaemia L1210 cell line was maintained as a suspension culture in RPMI-1640 medium supplemented with 10% fetal calf serum, glutamine (2 mM) and antibiotics (penicillin, 100 U $ml^{-1}$; streptomycin, 100 μg $ml^{-1}$). Hepes and sodium bicarbonate were added at final concentrations of 18 mM and 11 mM respectively. Cell densities were routinely maintained between $1 \times 10^4$ and $8 \times 10^5$ $ml^{-1}$.

Growth inhibition experiments were used to assess the cytostatic effects of the compounds. Cells were seeded at $1 \times 10^4$ $ml^{-1}$ in triplicate in 24-well multidishes. After 24 h drugs were added in the combinations and at the concentrations specified in the figure legends. At this time one set of replicates was counted using a Coulter counter ($N_0$). After 48 h the remaining samples were counted ($N_t$). The percentage growth inhibition of drug-treated samples was estimated as $N_t - N_0$ (drug treated)/$N_t - N_0$ (control) × 100. In drug combination experiments, in which evidence of synergistic effects on cell growth or clonogenicity (see below) was being sought, the single, fixed concentration drug sample was taken as the control value ($N_t$ control) in the above equation.

Clonogenic survival assays were used to assess the cytotoxicity of the compounds. They were performed as previously described (Sebolt-Leopold and Scavone, 1992), except that colonies were counted by eye on a gridded light box. The drug treatment protocols are described in the figure legends, and were carried out in suspension culture before plating the cells in agar, in the absence of drugs, to estimate survival. Survival and growth inhibition curves show the mean of three independent experiments ± s.e. Where error bars are not displayed in the figures, it is because they are obscured by the symbols.

### *PADPRP assays*

PADPRP activity was measured in a permeabilised cell assay. L1210 cells were rendered permeable to exogenous [$^{32}$P]NAD by exposure to hypotonic buffer and cold shock, as described by Halldorsson *et al.* (1978). In order to reveal total available enzyme activity, a palindromic dodecanucleotide, which forms a short double-stranded hairpin loop with a blunt end demonstrated to activate PADPRP (Grube *et al.*, 1991), was included in the assay at a concentration of 20 μg $ml^{-1}$. Following incubation of the permeabilised cells with [$^{32}$P]NAD, incorporation of $^{32}$P into acid precipitable counts was estimated. The results are expressed as percentage activity of the drug-treated relative to the control samples, and are the mean of quadruplicate samples ± s.e.

### *NAD assays*

Cellular NAD levels were determined by a modification of the method of Nisselbaum and Green (1969). Cells were treated with drugs at the concentrations and for the times specified in the figure legends. Approximately $5 \times 10^6$ cells per sample were harvested at 4°C, washed once with ice-cold phosphate-buffered saline (PBS) and repelleted. The pellet was resuspended in 1.0 ml of 50% (v/v) ethanol and sonicated for 20 s. An aliquot was removed for protein estimation (Bradford, 1976), and then the suspension was centrifuged for 2 min in a microfuge. The supernatant liquid was used for NAD assays as described. Results are expressed as pmol NAD $mg^{-1}$ protein, and represent the average of three independent samples ± s.e.

*DNA strand break assays*

DNA strand-break levels were assessed using the technique of Kohn *et al.* (1981). Cells were prelabelled with 0.4 $\mu$Ci ml$^{-1}$ [$^{14}$C]TdR for 24 h, followed by a 6 h chase in non-radioactive medium. Cells were then exposed to drugs at the concentrations and for the times specified in the figure legends. Internal standards were similarly labelled with 1 $\mu$Ci ml$^{-1}$ [$^{3}$H]TdR, exposed to 300 cGy, then loaded on the same filters as the drug treated samples, and eluted at pH 12.2. To summarise the data obtained, the results were expressed using the 'relative elution' (RE) formula of Fornace and Little (1977). RE represents the amount of DNA from the treated samples retained on the filter as a ratio of control (untreated). It is calculated using (log $RR_{sample}$) – (log $RR_{control}$), where RR (relative retention) is the fraction of sample DNA retained on the filter when 50% of the internal standard DNA has eluted. Points represent six replicates from three individual experiments ± s.e.

**Figure 2** Effect of inhibitors on PADPRP activity in a permeabilised cell assay. Results are expressed as percentage inhibition of enzyme activity in the presence of increasing concentrations of inhibitors. (◆) PD 128763; (○) NU1025; (□) BZ; (▲) 3AB.

### Results

*PADPRP assays*

The relative potencies of the four compounds studied as inhibitors of PADPRP are shown in Figure 2, in which percentage PADPRP inhibition is plotted against compound concentration. The $IC_{50}$ values of PD 128763 and NU1025 in this *in vitro* assay were $0.36 \pm 0.01$ and $0.44 \pm 0.13$ $\mu$M respectively. 3AB and BZ were more than an order of mag-

**Figure 3** The effect of increasing concentrations of PADPRP inhibitors alone (●) or in conjunction with a fixed (100 $\mu$M) concentration of TM (□) on cell growth. (a) 3AB. (b) BZ (c) NU1025. (d) PD 128763.

nitude less potent, with $IC_{50}$ values of 19.1 ± 5.9 μM and 13.7 ± 6.9 μM. This approximately 50-fold decrease in the $IC_{50}$ value of PD 128763 compared with 3AB is in excellent agreement with the data of Suto *et al.* (1991).

*Growth inhibition assays*

The cytostatic effects of PADPRP inhibitors used alone or in conjunction with a fixed concentration (100 μM) of TM were investigated (Figure 3). Exposure of cells to TM alone caused inhibition of cell growth, with an $IC_{50}$ value of 361 ± 25 μM (results not shown). Co-exposure of cells to 100 μM TM with

**Table I** Comparison of the $IC_{50}$ values of the PADPRP inhibitors alone or in conjunction with 100 μM TM estimated from the growth inhibition experiments

| *Inhibitor* | *$IC_{50}$ (mM) ± s.e. inhibitor alone* | *$IC_{50}$ (mM) ± s.e. inhibitor + 100 μM TM* |
|---|---|---|
| 3-Aminobenzamide | 6.7 ± 0.2 | 2.5 ± 0.1 |
| Benzamide | 2.5 ± 0.3 | 0.84 ± 0.12 |
| NU1025 | 0.41 ± 0.06 | 0.04 ± 0.003 |
| PD 128763 | 0.45 ± 0.01 | 0.023 ± 0.002 |

The $IC_{50}$ values were derived from the smooth curve analysis of GraphPad Inplot, San Diego, CA, USA software and were averaged from at least three independent experiments ± s.e.

increasing concentrations of PADPRP inhibitors caused a synergistic increase in growth inhibition (Figure 3). Note that for these experiments, the growth of cells in 100 μM TM, which itself reduced growth by about 26%, has been normalised to 100% (see Materials and methods). The $IC_{50}$ values for the inhibitors alone or in conjunction with 100 μM TM are summarised in Table I.

Ten to 20-fold higher concentrations of PD 128763 and NU1025 alone were required to inhibit cell growth than were required when the compounds were used in conjunction with 100 μM TM. For example, the $IC_{50}$ of NU1025 alone was 0.41 mM, and this was reduced to 0.04 mM in the presence of TM. In comparison, only 2- to 3-fold differences were obtained with 3AB and BZ, where there was considerable overlap between the growth-inhibitory effects of the compounds *per se*, and their effects in conjunction with TM. The potency of the compounds as PADPRP inhibitors reflected their effectiveness as inhibitors of cell growth, although this does not constitute proof that PADPRP function is essential for cell growth.

*Clonogenic survival assays*

It was necessary to establish that growth inhibition actually reflected cytotoxicity. Clonogenic survival assays were performed, where cells were exposed to increasing concentra-

**Figure 4** The effect of a 16 h exposure of cells to increasing concentrations of TM, in the presence or absence of fixed concentrations of PADPRP inhibitors, on clonogenic survival (**a**) ●, Control; □, + 1 mM 3AB; ▲, + 5 mM 3AB. (**b**) ●, control; □, + 1 mM BZ; ▲, + 3 mM BZ. (**c**), ●, control; □, + 10 μM NU1025; Δ, + 50 μM NU1025; ◆, + 100 μM NU1025. (**d**) ●, control; □, + 10 μM PD 128763; Δ, + 50 μM PD 128763; ◆, + 100 μM PD 128763.

tions of TM for 16 h, either alone or in the presence of fixed concentrations of PADPRP inhibitors, before plating for survivors in the absence of drugs. The survival curves are presented in Figure 4, and the $DEF_{10}$ values given in Table II. ($DEF_{10}$ is the ratio of the concentration of TM that reduces survival to 10% divided by the concentration of TM that reduces survival to 10% in the presence of a fixed concentration of PADPRP inhibitor). It can be seen that there was a reasonable correlation between growth inhibitory

**Table II** Comparison of the $DEF_{10}$ values obtained for a range of concentrations of the PADPRP inhibitors derived from the clonogenic assays shown in Figure 4

| *Inhibitor* | *Concentration* | *$DEF_{10}$*[a] |
|---|---|---|
| 3-Aminobenzamide | 1 mM | 2.4 ± 0.3 |
| | 5 mM | 4.1 ± 0.4 |
| Benzamide | 1 mM | 4.0 ± 0.7 |
| | 3 mM | 6.9 ± 0.2 |
| NU1025 | 10 μM | 2.0 ± 0.2 |
| | 50 μM | 4.0 ± 0.5 |
| | 100 μM | 5.1 ± 0.7 |
| PD128763 | 10 μM | 2.0 ± 0.1 |
| | 50 μM | 6.0 ± 0.5 |
| | 100 μM | 7.1 ± 0.4 |

$Def_{10}$ values were calculated using the smooth curve analysis described in Table I. Each value represents the average ± s.e. derived from the averaged 10% survival for TM alone (675 ± 31 μM from 22 independent survival curves) divided by individual 10% survival values from at least three independent survival curves performed in the presence of a fixed concentration of inhibitor.

**Figure 5** The effect of TM and the PADPRP inhibitors on the cellular NAD levels. (**a**) The effects of two fixed concentrations of TM was followed with time ●, 1 mM; ■, 2 mM. (**b**) The effect of increasing concentrations of PADPRP inhibitors on NAD levels in cells treated with 2 mM TM for 4 h incubation: ▲, 3AB; ◆, PD 128763.

and cytotoxic effects for TM alone with an $IC_{50}$ value of 361 μM ± 25 μM and a $LD_{50}$ value of 251 ± 13 μM, respectively, despite the differing exposure times (48 h for growth inhibition and 16 h for cytotoxicity). TM has a half-life in culture of about 40 min (Tsang *et al.*, 1991), and therefore will exert its full effects well before the minimum duration of exposure of either experiment. All compounds potentiated the cytotoxicity of TM, but PD 128763 and NU1025 produced about the same $DEF_{10}$ values at approximately 100-fold and approximately 60-fold lower concentrations than 3AB and BZ respectively (Table II). For example, 50 μM NU1025 and 5 mM 3AB gave equivalent $DEF_{10}$ values of approximately 4. For both PD 128763 and NU1025, maximal potentiation of cytotoxicity was obtained by concentrations of 50–100 μM, and was significant at doses as low as 10 μM.

The cytotoxic effects of the compounds alone were also investigated. The $LD_{50}$ values for a 24 h exposure were 14 ± 1.0 mM (3AB); 6.0 ± 1.5 mM (BZ); 1.6 ± 0.1 mM (NU1025) and 0.99 ± 0.18 mM (PD 128763) (results not shown). The $LD_{50}$ values differed by $\leqslant$ 3-fold from the $IC_{50}$ values, and again reflected their potency as PADPRP inhibitors. In agreement with the growth inhibition data there was a $\geqslant$ 10-fold difference between the concentrations of PD 128763 and NU1025 required to produce maximal potentiation of TM cytotoxicity and the concentrations required to produce cytotoxicity *per se*.

*NAD assays*

Changes in NAD levels are a convenient, albeit indirect, assessment of PADPRP activation in TM-treated cells. Figure 5a shows a time-dependent depletion of NAD levels following treatment with 1 or 2 mM TM. Evidence that the NAD depletion is mediated by PADPRP activation is shown in Figure 5b. A 4 h incubation with 2 mM TM caused a 50% decrease in cellular NAD levels, and this was abrogated in a concentration-dependent manner by PD 128763 and 3AB. Note that 10 μM PD 128763 sufficed to prevent approximately 50% of NAD drop, and that NAD depletion was completely prevented by 100 μM. These data correlate with the concentration ranges of PD 128763 required to effect potentiation of cytotoxicity in the clonogenic survival experiments. In contrast, at least an order of magnitude higher concentrations of 3AB were required to exert the same effects on NAD levels in TM-treated cells.

*DNA strand break assays*

The effect of the PADPRP inhibitors on DNA strand break levels in TM-treated cells was monitored by alkaline elution. A 1 h treatment with TM resulted in a concentration-dependent increase in the rate of elution (results not shown). Changes in DNA strand break levels were detectable at levels of TM as low as 150 μM, which reduced survival by about 30%. All the compounds were tested for their ability to produce strand breaks when used alone. A 24 h incubation of cells with 1 mM PD 128763 or NU1025 and 20 mM 3AB or BZ had no effect on DNA strand-break levels compared with untreated cells (results not shown).

Co-incubation of a fixed concentration of TM (150 μM) with increasing concentrations of all PADPRP inhibitors for 1 h caused a progressive increase in the rate of elution compared with TM alone. A specimen elution profile for the effect of increasing concentrations of NU1025 on TM-induced DNA strand break levels is shown in Figure 6. The results for all four compounds have been summarised by plotting RE values *vs* inhibitor concentration, and are shown in Figure 7. Note that the RE values for TM + inhibitor-treated cells have been calculated using TM alone controls, and not untreated cells. For all the compounds, the RE value increased linearly with increasing concentration. However, RE values started increasing for PD 128763 and NU1025 at about 100 μM, whereas concentrations above 3 mM and 5 mM were required to increase significantly the RE values for BZ

and 3AB respectively. Again, the potency of the compounds in the DNA strand break assay demonstrated an excellent correlation with *in vitro* PADPRP inhibitory potency.

Finally, the temporal kinetics of TM-induced DNA strand-break formation and religation was analysed, and the results are presented in Figure 8. In cells treated with 200 μM TM, DNA strand-break levels increased rapidly up to 4 h and declined thereafter. By 24 h DNA strand break levels had returned to almost control levels. Both 3AB (5 mM) and NU1025 (300 μM) increased net levels of DNA strand breaks over the entire time period. The time interval during which DNA strand break levels were highest (approximately 2–4 h) correlated with the reported timing of the peak levels of MTIC obtained in culture medium following addition of TM (Tsang *et al.*, 1991). This implies that the breakdown of MTIC to the methyl diazonium ion, which directly alkylates DNA, is relatively rapid compared to the decomposition of TM to MTIC in culture medium. It should be emphasised that these data do not differentiate between enhanced incision or reduced ligation as a causative mechanism for the net increase in DNA strand break levels observed in inhibitor-treated cells.

**Figure 6** DNA strand-break levels assessed by alkaline elution. The effect of co-incubation with increasing concentrations of NU1025 in cells treated with a fixed concentration (150 μM) of TM for 1 h. ●, Control (untreated); ■, TM alone; O, TM + 0.3 mM NU1025; Δ, TM + 0.5 mM NU1025; □, TM + 1.0 mM NU1025.

**Figure 7** The effect of increasing concentrations of PADPRP inhibitors on DNA strand-break frequency in cells treated with 150 μM TM for 1 h. In this case RE values have been calculated as a ratio of the RR values for PADPRP inhibitor-treated cells over the RR value of TM-treated cells. RE values have been plotted against increasing inhibitor concentration. ◆, PD 128763; O, NU1025; □, BZ; ▲, 3AB.

### Discussion

This is the first report of a comprehensive and quantitative analysis comparing the effects of a range of PADPRP inhibitors on PADPRP activity and on the biological end points associated with the cellular responses to DNA damage. Suto *et al.* (1991) and Sebolt-Leopold and Scavone (1992) demonstrated that PD 128763 potentiated the cytotoxicity of ionising radiation, the monofunctional alkylating agent, streptozotocin, and also 2-nitroimidazole. However, PD 128763 was only used at a concentration of 500 μM in their experiments, thereby potentially underestimating the potency of this compound in intact cells. Here we have established that maximal potentiation of TM is obtained at a 10-fold lower concentration (50 μM), and is significant at concentrations as low as 10 μM. However, we cannot rule out the possibility that the concentration-dependent effects of PADPRP inhibitors may vary with different DNA-damaging agents, and in different cell lines.

In phase I trials, following a dose of TM (200 mg m$^{-2}$), peak plasma levels of approximately 50 μM were achieved by 2 h (Newlands *et al.*, 1992). These levels are of the same order of magnitude as the concentration (⩾ 100 μM) used in our experiments, in which potentiation of cytotoxicity by PADPRP inhibitors was observed.

The new compounds are about 50-fold more potent as PADPRP inhibitors than 3AB and BZ, and this differential is

**Figure 8** The effect of 200 μM TM ± PADPRP inhibitors on DNA strand-break levels over a 24 h time course. RE values have been plotted against time (**a**) ●, 200 μM TM alone; Δ, 200 μM TM + 5 mM 3AB. (**b**) ●, 200 μM TM alone; □, 200 μM TM + 300 μM NU1025. Single data points are shown from representative experiments, where TM ± PADPRP inhibitor treatments were carried out in parallel.

maintained in intact cells when the cellular responses to DNA damage known to be modulated by PADPRP function (i.e. cytotoxicity and DNA repair) are investigated. Furthermore, there is more than an order of magnitude difference in the concentration of PD 128763 and NU1025 (10–50 μM) required to potentiate TM cytotoxicity compared with the concentrations of the compounds alone required to exert cytotoxic effects (⩾ 1 mM). In comparison, considerable overlap is evident in the concentrations of 3AB and BZ required to exert these two effects. Thus, there is a bigger gap between the synergistic enhancement of cytotoxicity and the independent toxicity of the new inhibitors compared with 3AB and BZ, indicative of improved specificity.

It has not escaped our notice that the data presented here suggest a dissociation between the effect of PADPRP inhibitors on cell survival and their effect on DNA strand break repair. For example, significant potentiation of TM cytotoxicity is obtained with PD 128763 at 10 μM, and has reached its maximum by 50 μM (see Figure 4). In contrast, PD 128763 does not affect TM-induced DNA strand break levels significantly until concentrations of ⩾ 100 μM are reached (Figure 7). A plausible explanation for these observations is that PADPRP mediates not only DNA repair processes, but also independently modulates DNA damage-inducible responses involved in cell survival (e.g. specific gene transcription, p53 stabilisation; Kastan *et al.*, 1991). The difference in the concentrations of inhibitors required would reflect the degree of inhibition of PADPRP necessary to modulate these responses. A more detailed analysis of these observations is currently under way.

In conclusion, PD 128763 and NU1025 can potentiate the cytotoxicity of clinically relevant concentrations of TM, and at micromolar compared with millimolar concentrations required for BZ and 3AB. These are important considerations if the use of these compounds as potentiators of drug-induced cytotoxicity is to be extrapolated to the clinic. These data provide the groundwork for initiating *in vivo* studies to establish the ability of PADPRP inhibitors, used in conjunction with chemotherapeutic agents, to enhance tumour regression.

### Abbreviations

3AB, 3-aminobenzamide; BZ, benzamide; $IC_{50}$, concentration which reduces growth/activity by 50%; DMSO, dimethyl sulphoxide; DBD, DNA-binding domain; $DEF_{10}$, dose enhancement factor at 10% survival; $LD_{50}$, dose which reduces survival by 50% (lethal dose); MTIC, 5-(3-methyl-triazen-l-yl) imidazole-4-carboxamide; PBS, phosphate-buffered saline; PADPRP, poly(ADP-ribose) polymerase; RE, relative elution; RR, relative retention; TM, temozolomide; s.e., standard error.

### Acknowledgements

We would like to express our gratitude to Professor MFG Stevens, University of Nottingham, for the provision of temozolomide, and to Dr WR Leopold, Warner Lambert, for the provision of PD 128763. Our thanks to Professor DR Newell, Cancer Research Unit, University of Newcastle upon Tyne, for invaluable discussion and advice. Our thanks also to Karen Bowman for performing some of the PADPRP assays.

### References

ALTHAUS FR, HOFFERER L, KLECZKOWSKA HE, MALANAGA M, NAEGELI H, PANZETER P AND REALINI C. (1993). Histone shuttle driven by the automodification of poly(ADP-ribose) polymerase. *Environ. Mol. Mutagen.*, **22**, 278–282.

BANASIK M, KOMURA H, SHIMOYAMA M AND UEDA K. (1992). Specific inhibitors of poly(ADP-ribose) synthetase and mono(ADP-ribosyl) transferase. *J. Biol. Chem.*, **267**, 1569–1575.

BOULIKAS T. (1991). Relation betweeñ carcinogenesis, chromatin structure and poly(ADP-ribosylation) (review). *Anticancer Res.*. **11**, 489–528.

BRADFORD MM. (1976). A rapid and sensitive method for the quantitiation of microgram quantities of protein utilizing the principle of protein-dye binding. *Anal. Biochem.*, **72**, 248–254.

CHATTERJEE S, CHENG MF, BERGER SJ AND BERGER NA. (1991). Alkylating agent hypersensitivity in poly(adenosine diphosphate-ribose) polymerase deficient cell lines. *Cancer Commun.*, **3**, 71–75.

CREISSEN D AND SHALL S. (1982). Regulation of ligase activity by poly(ADP-ribose). *Nature*, **296**, 271–272.

DE MURCIA G AND MÉNISSIER DE MURCIA J. (1994). Poly(ADP-ribose) polymerase: a molecular nick-sensor. *Trends Biol. Sci.*, **19**, 172–176.

DENNY BJ, WHEELHOUSE RT, STEVENS MFG, TSANG LH AND SLACK JA. (1994). NMR and molecular modeling investigation of the mechanism of activation of the antitumor drug temozolomide and its interaction with DNA. *Biochemistry*, **33**, 9045–9051.

DURKACZ BW, OMIDIJI O, GRAY DA AND SHALL S. (1980). (ADP-ribose)$_n$ synthesis participates in DNA excision repair. *Nature*, **283**, 593–596.

FERRO AM, HIGGINS NP AND OLIVERA BM. (1983). Poly(ADP-ribosylation) of a DNA topoisomerase. *J. Biol. Chem.*, **258**, 6000–6003.

FORNACE JR AJ AND LITTLE JB. (1977). DNA crosslinking induced by X-rays and chemical agents. *Biochim. Biophys. Acta*, **477**, 343–355.

GRIFFIN RJ, PEMBERTON LC, RHODES D, BLEASDALE C, BOWMAN K, CALVERT AH, CURTIN NJ, DURKACZ BW, NEWELL DR, PORTEOUS JK AND GOLDING BT. (1995). Novel potent inhibitors of the DNA repair enzyme poly(ADP-ribose) polymerase (PARP). Anticancer Drug Design (in press).

GRUBE K, KÜPPER JH AND BÜRKLE A. (1991). Direct stimulation of poly(ADP-ribose) polymerase in permeabilised cells by double-stranded DNA oligomers. *Anal. Biochem.*, **193**, 236–239.

HALLDORSSON H, GRAY DA AND SHALL S. (1978). Poly(ADP-ribose) polymerase activity in nucleotide permeable cells. *Febs Lett*, **85**, 349–352.

HUNTING DJ, GOWANS BJ AND HENDERSON JF. (1985). Specificity of inhibitors of poly(ADP-ribose) synthesis. Effects of nucleotide metabolism in cultured cells. *Mol. Pharmacol.*, **28**, 200–206.

KASTAN MB, ONYEKWERE O, SIDRANSKY D, VOGELSTEIN B AND CRAIG RW. (1991). Participation of p53 protein in the cellular response to DNA damage. *Cancer Res.*, **51**, 6304–6311.

KOHN KW, EWIG RAG, ERICKSON LC AND ZWELLING LA. (1981). Measurement of strand breaks and crosslinks by alkaline elution. In: *DNA Repair: A Laboratory Manual of Research Procedures*, Vol. 1, part B, Friedberg EC and Hanawalt PC (eds). pp. 379–401. Marcel Dekker: New York.

LEOPOLD WR AND SEBOLT-LEOPOLD JS. (1992). Chemical approaches to improve radiotherapy. In: *Cytotoxic Anticancer Drugs: Models and Concepts for Drug Discovery and Development*, Valeriote FA, Corbett TH anmd Baker LH (eds), chapter 9. Kluwer Academic Publishers: Boston.

MACLAREN RA, WITMER MV, RICHARDSON E AND STAMATO TD (1990). Isolation of Chinese hamster ovary cells with reduced poly(ADP-ribose) polymerase activity. *Mutat. Res.*, **231**, 265–274.

MOLINETTE M, VERMEULEN W, BÜRKLE A, MÉNISSIER-DE MURCIA J, KÜPPER JH, HOEIJMAKERS JHJ AND DE MURCIA G. (1993). Overproduction of the poly(ADP-ribose) polymerase DNA-binding domain blocks alkylation-induced DNA repair synthesis in mammalian cells. *EMBO J.*, **12**, 2109–2117.

MOSES K, WILLMORE E AND DURKACZ BW. (1990). Correlation of enhanced 6-mercaptopurine cytotoxicity with increased phosphoribosylpyrophosphate levels in Chinese hamster ovary cells treated with 3-aminobenzamide. *Cancer Res.*, **50**, 1992–1996.

NEWLANDS ES, BLACKLEDGE GRP, SLACK JA, RUSTIN GJS, SMITH DB, STUART NSA, QUARTERMAN CP, HOFFMAN R, STEVENS MFG, BRAMPTON MH AND GIBSON AC. (1992). Phase I trial of temozolomide (CCRG 81045: M & B 39831: NSC 362856). *Br. J. Cancer*, **65**, 287–291.

NISSELBAUM JS AND GREEN S. (1969). A simple ultramicro method for the determination of pyridine nucleotides in tissue. *Anal. Biochem.*, **27**, 212–217.

PURNELL MR AND WHISH WJD. (1980). Novel inhibitors of poly(ADP-ribose) synthetase. *Biochem. J.*, **185**, 775–777.

RANKIN PW, JACOBSON EL, BENJAMIN RC, MOSS J AND JACOBSON MK. (1989). Quantitative studies of inhibitors of ADP-ribosylation *in vitro* and *in vivo*. *J. Biol. Chem.*, **264**, 4312–4317.

SATOH MS AND LINDAHL T. (1992). Role of poly(ADP-ribose) formation in DNA repair. *Nature*, **356**, 356–358.

SATOH MS, POIRIER GG AND LINDAHL T. (1993). $NAD^+$-dependent repair of damaged DNA by human cell extracts. *J. Biol. Chem.*, **268**, 5480–5487.

SEBOLT-LEOPOLD JS AND SCAVONE SV. (1992). Enhancement of alkylating agent activity *in vitro* by PD 128763, a potent poly(ADP-ribose) synthetase inhibitor. *Int. J. Radiat. Oncol. Biol. Phys.*, **22**, 619–621.

SMULSON M, ISTOCK N, DING R AND CHERNEY B. (1994). Deletion mutants of poly(ADP-ribose) polymerase support a model of cyclic association and dissociation of enzyme from DNA ends during DNA repair. *Biochemistry*, **33**, 6186–6191.

STEVENS MFG, HICKMAN JA, LANGDON SP, CHUBB D, VICKERS L, STONE R, BAIG G, GODDARD C, GIBSON NW, SLACK JA, NEWTON C, LUNT E, FIZAMES C AND LAVELLE F. (1987). Antitumour activity and pharmacokinetics in mice of 8-carbamoyl-3-methyl-imidazo[5,1-*d*]-1,2,3,5-tetrazin-4(3*H*)-one (CCRG81045; M & B 39831), a novel drug with potential as an alternative to decarbazine. *Cancer Res.*, **47**, 5846–5852.

SUTO MJ, TURNER WR, ARUNDEL-SUTO CM, WERBEL LM AND SEBOLT-LEOPOLD JS. (1991). Dihydroisoquinolinones: the design and synthesis of a new series of potent inhibitors of poly(ADP-ribose) polymerase. *Anti-cancer Drug Design*, **7**, 101–107.

TSANG LLH, QUARTERMAN CP, GESCHER A AND SLACK JA. (1991). Caomparison of the cytotoxicity in vitro of temozolomide and decarbazine, prodrugs of 3-methyl-(triazen-1-yl)imidazole-4-carboxamide. *Cancer Chemother. Pharmacol.*, **27**, 342–346.

WITMER MV, ABOUL-ELA N, JACOBSON ML AND STAMATO TD. (1994). Increased sensitivity to DNA-alkylating agents in CHO mutants with decreased poly(ADP-ribose) polymerase activity. *Mutat. Res. DNA Repair*, **314**, 249–260.

YOSHIHARA K, ITAYA A, TANAKA Y, OHASHI Y, ITO K, TEAOKA H, TSUKADA K, MATSUKAGE A AND KAMIYA T. (1985). Inhibition of DNA polymerase α, DNA polymerase β, terminal deoxynucleotidyltransferase and DNA ligase by poly(ADP-ribosyl)ation in vitro. *Biochem. Biophys. Res. Commun.*, **128**, 61–67.